# Returning genome sequences to research participants: Policy and practice

**DOI:** 10.12688/wellcomeopenres.10942.1

**Published:** 2017-02-24

**Authors:** Caroline F. Wright, Anna Middleton, Jeffrey C. Barrett, Helen V. Firth, David R. FitzPatrick, Matthew E. Hurles, Michael Parker

**Affiliations:** 1 Wellcome Trust Sanger Institute, Wellcome Genome Campus, Hinxton, Cambridge, CB10 1SA, UK; 2 East Anglian Medical Genetics Service, Cambridge University Hospitals NHS Foundation Trust, Cambridge Biomedical Campus, Cambridge, CB2 0QQ, UK; 3 MRC Human Genetics Unit, MRC IGMM, University of Edinburgh, Western General Hospital, Edinburgh, EH4 2XU, UK; 4 The Ethox Centre, Nuffield Department of Population Health, University of Oxford, Oxford, OX3 7LF, UK

**Keywords:** Data sharing, genomics, ethics, sequencing, incidental findings, DDD study

## Abstract

Despite advances in genomic science stimulating an explosion of literature around returning health-related findings, the possibility of returning entire genome sequences to individual research participants has not been widely considered. Through direct involvement in large-scale translational genomics studies, we have identified a number of logistical challenges that would need to be overcome prior to returning individual genome sequence data, including verifying that the data belong to the requestor and providing appropriate informatics support. In addition, we identify a number of ethico-legal issues that require careful consideration, including returning data to family members, mitigating against unintended consequences, and ensuring appropriate governance. Finally, recognising that there is an opportunity cost to addressing these issues, we make some specific pragmatic suggestions for studies that are considering whether to share individual genomic datasets with individual study participants. If data are shared, research should be undertaken into the personal, familial and societal impact of receiving individual genome sequence data.

## Context

**Figure 1.**
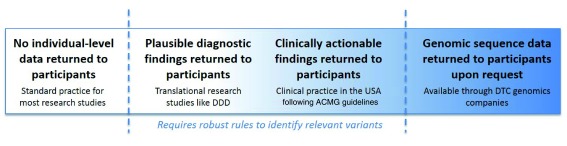
Options for returning genome sequence data to participants. DDD, Deciphering Developmental Disorders study; ACMG, American College of Medical Genetics and Genomics; DTC, direct-to-customer.

As DNA sequencing becomes cheaper and more widely available, the question of what genomic data could or should be returned to an individual research participant has become important ( Figure 1). To date, most discussion in the literature has focused on the relative merits of returning specific health-related findings that are either pertinent or incidental to the purpose of sequencing ^1^. Strong arguments have been made both for and against returning actionable findings ^2– 4^, and numerous clinical and research laboratories have developed robust systems for identifying, assessing and reporting variants that might fall into this category ^5^.

The option of returning individual ‘raw’ genome sequence data – BAM or VCF files (see Table 1) – has thus far received less attention, and until now the best practice has been not to share sequence data with individual participants ^6^. Since the vast majority of the public (and indeed most researchers) would be unable to meaningfully interpret large and complex genomic datasets, the value of returning such data is unclear. However, although there is currently no legal avenue for participants to request their data ^7^, and no expectation upon researchers to share data if it negatively affects their research ^8^, the public are widely supportive of the idea and anecdotal examples exists of such requests being made ^9^. When surveyed, most people indicate that they plan to seek out an interpretation of their data, while others wish to access their data simply because it is ‘theirs’ ^9^. Coupled with the potential benefits – supporting autonomy, empowerment, health prevention, reciprocity, and improved trust between researchers and society ^10^ – this suggests researchers and research institutions have an obligation to consider whether, how and specifically what individual sequence data could reasonably be returned to study participants. Moreover, as genome sequencing becomes more integrated into mainstream healthcare, scientists may come under increasing pressure to accede to requests to provide individual research participants with their data. Anticipating this development, the UK 100,000 Genomes Project Protocol (Genomics England, January 2017, Section 9.1.1; https://www.genomicsengland.co.uk/information-for-gmc-staff/rare-disease-documents/) already states that “ *patients will have the right to request that their whole genome sequencing (WGS) data be made available to them.*”

**Table 1.** Genomic sequence data file types and sizes. *GB, gigabytes; WES, whole exome sequencing (i.e. all the protein coding regions of the genome, ~50–60 million bases); WGS, whole genome sequencing (~3 billion bases, assumes 30X coverage).

Name	Description	WES file size (GB)	WGS files size (GB)
BAM	*Binary Alignment/ Map format*: nucleotide sequence with corresponding quality scores mapped to the reference genome, derived from raw data files using an alignment algorithm	~5–15	~150–250
VCF	*Variant Call Format*: files for storing variant bases relative to the reference genome, which are derived from sequence files using a variant calling algorithm; usually annotated with allele frequency and predicted consequence	~0.02	~0.2

Given that such requests are likely to increase, here we outline the issues that need to be considered when formulating a policy on returning genome sequence data to individual study participants. Specifically, we address two questions: what are the resource implications of returning sequence data and, if it were cost-neutral (or specifically funded within the research proposal), should a mechanism be provided to facilitate individual-level data sharing?

As a case study to illuminate the issues, we use the UK Deciphering Developmental Disorders (DDD) Study, a translational research project involving whole exome sequencing of nearly 14,000 clinically-ascertained families (>32,000 exome sequences) to find the genetic causes of severe developmental disorders ^11^. The DDD study has pioneered a proportionate data sharing policy ^12^, which returns likely diagnostic variants to families via DECIPHER ^13^ and their local clinical teams, and enables *bona fide* researchers to access anonymised whole exome sequence data from the European Genome-phenome Archive under a managed access model ^14^. However, the DDD study does not currently return whole exome sequence data to individual participants, a decision that was reached after careful consideration of the issues outlined below and how best to deploy the study’s finite resources.

## Logistical challenges

Even in cases such as the DDD study, where exome sequencing data have been generated and there is a direct link (via a clinician) to each individual participant, there are still substantial resources required to ensure that whole exome data could be safely returned to individuals ^7^.

*Confirming the data belong to the requestor*: the laboratory and data management systems utilised by researchers are not generally required to meet the rigorous quality standards of accredited diagnostic laboratories. Therefore, it is imperative to confirm that any data generated through a research pipeline actually belongs to the individual requesting the data. Sharing person A’s unique genomic data with person B, without their knowledge or consent, would be a negligent and egregious abuse of trust and, depending on the use to which the data were put, could constitute a major clinical risk. To solve this problem, a second DNA sample could be compared to the dataset using a DNA fingerprint. Resources would be needed to support secondary sampling, alongside processes to manage sample logistics, DNA extraction, genotyping and data analysis.

*Providing bespoke informatics support:* although some research participants may have some degree of personal bioinformatics expertise, who may request their data for a variety of reasons, providing the data in an accessible format would likely require some informatics support. Research datasets are frequently generated and annotated using experimental bioinformatics pipelines that are constantly evolving, and annotations can change substantially over time. Additionally, care must be taken to remove annotations common in such files that might reveal sensitive information about other members of the cohort. It would therefore be necessary to develop a specific bioinformatics pipeline to clean any research datasets prior to release to participants. Data return options vary from a simple encrypted hard drive to a user-friendly secure data access portal, which would require substantial resources to set-up and manage, but would offer greater equity of access. Either option would require some technical management to ensure that the correct data are uploaded and accessed by the correct individual.

## Ethico-legal considerations

In addition to the resource-dependent issues identified above, there are a number of other issues that need to be considered in studies involving patient populations, such as families in the DDD study.

*Returning data to family members:* since the majority of genetic variants are shared between close biological relatives, any returned data may have implications for other (un-sequenced) members of the family ^15^. Although a single participant may have a right to their own genome data, regardless of whether it is "shared" with family members, the familial implications should be made clear, and in cases where an entire family’s data are requested, consent should be sought from all individuals or proxies upon whom data are being requested. In addition, paediatric studies, such as DDD, must address the thorny issue of returning a child’s genome sequence to their parents ^16^, where it is complex to determine who has rights of control and to what extent as the child approaches and passes the age of majority. There are already well rehearsed arguments that attempt to balance an individual’s right to privacy with the desire to protect their relatives from harm ^15^, but this issue is even more complex within the context of a long-term research study, during which time – unbeknownst to the researchers – an individual may gain (or lose) the capacity to make a decision about their own data. Researchers should be aware of the legal status of the data within their jurisdiction, particularly in relation to individuals who lack capacity to give consent.

*Mitigating against unintended consequences:* much of the previous literature discussing return of individual results from genome sequencing studies has focused on the utility of the data and its limitations ^17– 19^. Our empirical data on attitudes towards the return of genome sequence data has shown that while research participants say they would like access to their ‘raw data’, many have unrealistic expectations of what they can do with it, e.g. 43% (n=1844) said they would take their raw DNA data to their General Practitioner ^9^. Although numerous genome interpretation services exist, unlike returning specific health-related findings where the onus lies with the research team to develop robust processes for identifying and verifying relevant variants, researchers could not be held responsible for any participant-initiated or third party analyses of their data. Nonetheless, there are legitimate concerns about the moral and legal responsibilities of researchers and research institutes for any harm that might befall participants or members of their family as a result of data return. There are no regulated standards for genomic analysis and very limited consensus between existing interpretation services for personal whole genome data ^20^. There could also be a substantial impact on clinical services, due to interpretation requests and follow-on work, creating a significant and potentially unmanageable healthcare demand. Data that are analysed in a clinical context could potentially result in litigation for negligence due to a different interpretation of results, an incorrect diagnosis, or even a ‘missed’ life-saving diagnosis. To mitigate against these risks, the procedure for data generation and processing should follow best practice (to the extent that it is defined) and be clearly documented, along with known limitations as part of the terms and conditions of receiving data. Researchers must be clear that they are not providing a clinical service, but participants should be signposted to appropriate sources of further information to help them decide what to do next. Within the wider ecosystem, development of quality-assured (e.g. “kite-marked”) websites are needed, which both clinical geneticists and genomic scientists should agree are useful for the public.

*Ensuring appropriate governance:* careful consideration and oversight of the issues outlined above is essential for any study wishing to return genomic sequence data to individual participants. There needs to be a formal and transparent process in place for making decisions, which involves a number of stakeholders involved both in making the initial decision to disclose data and in maintaining on-going oversight and ensuring good data governance. This might include the principal investigators, clinical and research study sponsor(s), professional bodies (including legal and ethics representation) and patient representatives.

## Conclusions

In the context of the open data movement and the rapid growth of genome sequencing, it is likely that researchers will increasingly face requests from study participants to access their own data, for various reasons. Research studies could potentially face reputational risk, loss of trust or litigation either from withholding data without good reason, or from inappropriate or reckless data return. Researchers have a duty to society and their funders to make the best use of finite resources, and must balance their moral responsibilities to individual participants with broader responsibilities to the whole cohort and the creation of generalizable knowledge (note, these duties are often different to the duties of clinicians). Moreover, erroneous diagnoses arising from over-interpretation of the data and poor implementation of genomics could result in real harm to patients, particularly in translational studies involving vulnerable clinical groups. There is a pressing need for a rigorous public health evaluation of the significance of identifying actionable variants in asymptomatic individuals with no family history.

We conclude that unless the return of genomic sequence data to individual participants is specifically and appropriately resourced, potentially by research funders themselves, to do so could do more harm than good. How can we reconcile this with mounting ethical arguments in favour of data sharing, and strong empirical support for receiving individual genome sequence data? Where resources are available to support robust sample and data management ^21^, as well as good data governance, returning individual genome sequence data to research participants is logistically achievable and may also be morally desirable. For studies considering whether to share individual genomic datasets with individual study participants, we make the following pragmatic suggestions:

(1) A second sample should be obtained from all requestors to verify the provenance of the data;
(2) Informatics support should be provided to generate appropriately processed datasets and enable individuals to access their data;
(3) Research budgets should explicitly include funding to support return of individual-level data;
(4) The research team should document their protocols, processes and data limitations, and signpost participants to quality sources of information;
(5) Appropriate governance structures should be put in place to ensure data access requests are handled correctly, including signed consent forms from all requestors;
(6) Research should be undertaken into the personal, familial and societal impact of receiving individual genome sequence data.
