# Supplementary material for: Returning genome sequences to research participants: Policy and practice
Source: Wellcome Open Res. 2017 Feb 24;2:15. [Version 1] doi: 10.12688/wellcomeopenres.10942.1 (PMC5351846; doi:10.12688/wellcomeopenres.10942.1)
Supplement: Peer review history

# Peer review: Referee response for version 1

**DOI:** 10.21956/wellcomeopenres.11795.r20527

**Published:** 2017-03-09

**Authors:** Alison Hall

**Affiliations:** 1 PHG Foundation, Cambridge, UK

This excellent paper addresses the challenges that might be raised if entire genome sequences were released to individual research participants. I particularly like the fact that the authors have described the logistical and bioinformatics support in tandem with ethico-legal considerations. As the authors point out in the conclusions section of the paper, research funders and researchers will need to pro-actively plan for whole genome sequence release, well in advance of its taking place. It seems increasingly likely that policies will need to take account of this possibility - and this paper deserves to be widely read.

Title and abstract: apposite, clear and concise

Article Content: One very minor point could be added if the paper is amended: this would be to highlight in a few words in the ethico-legal considerations discussion that unintended consequences might include unexpected knowledge about familial relationships (such as mis-attributed paternity or consanguinity - especially if multiple genomes from trios are returned). 

Conclusions: A helpful set of recommendations to guide future practice.

I have read this submission. I believe that I have an appropriate level of expertise to confirm that it is of an acceptable scientific standard.

# Peer review: Referee response for version 1

**DOI:** 10.21956/wellcomeopenres.11795.r20667

**Published:** 2017-03-06

**Authors:** Bartha Maria Knoppers

**Affiliations:** 1 Centre of Genomics and Policy, Department of Human Genetics, Faculty of Medicine, McGill University, Montréal, QC, Canada

“Returning genome sequences to research participants: Policy and practice” is an excellent article! Approved as is

Title and Abstract: The title of the article and abstract are appropriate

Article Content: The “Logistical Challenges” section is a novel (necessary and practical!) addition to traditional ELSI discussions.

Conclusions: The conclusions highlighting the resource/infrastructure issues as fundamental to realizing a return of results policy – bravo!

 P.S. SVP put the words “….no legal avenue in the UK for participants…” – p. 2 Context section (2 ^nd^ parag, 3 ^rd^ sentence)

I have read this submission. I believe that I have an appropriate level of expertise to confirm that it is of an acceptable scientific standard.
